# The Anterior Insula and its Projection to the Prelimbic Cortex are Involved in the Regulation of 5-HT-Induced Itch

**DOI:** 10.1007/s12264-023-01093-y

**Published:** 2023-08-08

**Authors:** Juan Yao, Xuan Li, Guang-Yan Wu, Bing Wu, Jun-Hui Long, Pu-Jun Wang, Shu-Lei Liu, Jie Gao, Jian-Feng Sui

**Affiliations:** 1 Experimental Center of Basic Medicine, Army Medical University, Chongqing, 400038 China; 2 Department of Physiology, College of Basic Medical Sciences, Army Medical University, Chongqing, 400038 China; 3 Department of Dermatology, The 958th Army Hospital of the People’s Liberation Army, Chongqing, 400020 China; 4 State Key Laboratory of Trauma, Burns and Combined Injury, Army Medical Centre of the PLA, Institute of Surgery Research, Daping Hospital, Army Medical University, Chongqing, 400042 China

**Keywords:** Itch, Insular cortex, Prelimbic cortex, Conditioned place aversion, Pharmacogenetic inhibition

## Abstract

Itch is an unpleasant sensation that urges people and animals to scratch. Neuroimaging studies on itch have yielded extensive correlations with diverse cortical and subcortical regions, including the insular lobe. However, the role and functional specificity of the insular cortex (IC) and its subdivisions in itch mediation remains unclear. Here, we demonstrated by immunohistochemistry and fiber photometry tests, that neurons in both the anterior insular cortex (AIC) and the posterior insular cortex (PIC) are activated during acute itch processes. Pharmacogenetic experiments revealed that nonselective inhibition of global AIC neurons, or selective inhibition of the activity of glutaminergic neurons in the AIC, reduced the scratching behaviors induced by intradermal injection of 5-hydroxytryptamine (5-HT), but not those induced by compound 48/80. However, both nonselective inhibition of global PIC neurons and selective inhibition of glutaminergic neurons in the PIC failed to affect the itching-scratching behaviors induced by either 5-HT or compound 48/80. In addition, pharmacogenetic inhibition of AIC glutaminergic neurons effectively blocked itch-associated conditioned place aversion behavior, and inhibition of AIC glutaminergic neurons projecting to the prelimbic cortex significantly suppressed 5-HT-evoked scratching. These findings provide preliminary evidence that the AIC is involved, at least partially *via* aversive emotion mediation, in the regulation of 5-HT-, but not compound 48/80-induced itch.

## Introduction

Itch is an unpleasant and unique sensation that drives scratching behaviors to expel cutaneous biological threats [1–3]. However, treatment-resistant itch and itch-related diseases are common and devastating clinical challenges with profound impacts on patients. Despite its clinical importance, we still know little about the basic mechanisms underlying itch modulation. During the past decades, neuroscientists and dermatologists have gained a preliminary understanding of the processes of itch signal generation, transmission, and regulation at the peripheral [4–7] and spinal [8–11] levels. In recent years, an increasing number of studies have explored the cortical and subcortical mechanisms of itch regulation.

Functional imaging studies have shown that itch and scratching lead to the activation of multiple brain regions, such as the prefrontal cortex, motor cortex, supplementary motor area, somatosensory cortex, basal ganglia, and cerebellum [12–18], suggesting that itch involves multiple information-processing centers in the brain. Recently, several optogenetic and pharmacogenetic studies in animals have confirmed the crucial roles in itch modulation of some subcortical centers, including the ventral tegmental area (VTA) [19, 20] and the periaqueductal gray (PAG) [21, 22] in the midbrain, and the amygdala [23, 24]. At the cortical level, recent studies have shown that itch and other somatosensory sensations are multiplexed in the primary somatosensory cortex [25–27]; descending projections from the primary somatosensory cortex innervate and activate inhibitory interneurons in the dorsal horn to tonically inhibit itch signal transmission in the spinal cord [28]; projections from the anterior cingulate cortex (ACC) to the dorsomedial striatum selectively regulate histaminergic itch in mice [29]; the prelimbic cortex regulates itch processing by controlling attentional bias [30]; and the ACC and prelimbic cortex differentially modulate 5-hydroxytryptamine (5-HT)- and compound 48/80-induced itch [31]. However, the contributions of the IC, which has been widely acknowledged to be involved in diverse brain functions, such as somatic and visceral sensation, movement, emotion, and cognition, remain poorly understood in itch regulation.

The IC is located deep in the lateral sulcus of the brain and can be divided into the AIC and PIC according to structure and function. The insula is considered to be responsible for the integration of multimodal information of sensation and emotion, especially for the formation of individual consciousness in the internal perception state [32, 33]. In recent decades, research on the role of the IC in itch regulation has been limited to imaging studies.

Functional imaging studies have shown that the anterior and posterior parts of the patients’ insula are activated during itch stimulation [16–18]; the insula is more sensitive to itchy stimuli than to pain stimuli [12], and is activated during not only chemically-induced itching [12], but also visually-induced (contaminated) itching, and even imagined itching [17]; and the AIC receives internal sensory input mainly through the PIC and displays stronger bilateral activation during pruritus stimulation [12]. The excitation of insular neurons has been assumed to mediate itch sensation, itch-associated negative emotions, cognition [12–16], and the pleasure caused by scratching [34]. In addition, a recent morphological study of histamine- and chloroquine-induced itch in mice demonstrated that the early-immediate genes c-fos and p-ERK, which indicate cellular excitation, are highly expressed in insular neurons, while 5-HT neurons in the dorsal raphe nucleus with projections to the insula are activated during this process [35]. Together, these findings suggest that the insula is involved in the regulation of acute itch.

Unfortunately, the above findings were all parallel, rather than causal demonstrations, and merely suggested a correlation between the insula and itch. Direct experimental evidence to confirm the involvement and contribution of the IC in itch regulation remains to be elucidated. Using fiber recording and pharmacogenetic experiments, we reveal here that both AIC and PIC neurons were activated during acute itch processes, and indiscriminate inhibition of the activity of global AIC neurons, or selective inhibition of the activity of AIC glutaminergic neurons, reduced the scratching behavior induced by intradermal injection of 5-HT (a non-histaminergic pruritogen), but not compound 48/80 (a histamine-releasing agent); however, both nonselective and selective inhibition of global PIC neurons or glutaminergic neurons in the PIC failed to change the itching-scratching behavior induced by 5-HT or compound 48/80. In addition, pharmacogenetic inhibition of the AIC glutaminergic neurons effectively blocked itch-associated conditioned place aversion (CPA) behavior, and selective inhibition of glutaminergic projections from the AIC to the prelimbic cortex (PrL) decreased the scratch responses induced by 5-HT. These findings provide preliminary evidence that the AIC and its projection to the PrL are involved in the regulation of 5-HT-, but not compound 48/80-induced itch and itch-associated aversion, which advances our understanding of the circuit mechanisms of itch modulation in the brain.

## Materials and Methods

### Animals

Adult male Sprague-Dawley rats (300–350 g and 3–4 months old) were housed in standard cages with a cycle of 12 h light/dark and a stable temperature of 21–25°C. They were provided with food and water *ad libitum*. The Animal Care Committee of the Army Medical University approved this animal experimental protocol.

### Stereotaxic Surgeries and Injections

Animal surgeries and virus injections were performed according to similar procedures described previously [36–38]. Three percent pentobarbital sodium was administrated to anesthetize the rats (40 mg/kg, dissolved in saline, i.p.). After fixation in a stereotaxic apparatus, viruses were injected into the brain at 0.05 µL/min with a glass micropipette (tip diameter 10–20 µm). Then, the micropipette was left in place for 5 additional minutes and slowly withdrawn.

Traditionally, calcium/calmodulin-dependent protein kinase (CaMKIIα) is a marker for glutamatergic cells [39–42]. Thus, to target glutaminergic neurons, we used CaMKIIα as a promoter. For fiber photometry recording of the activity of glutaminergic AIC and PIC neurons during itch behaviors, rAAV2/9-CaMKIIα-jGCaMP6s (200 nL, titer: 5.90 × 10^12^ genome copies (GC)/mL), or rAAV2/9-CaMKIIα-EGFP (200 nL, titer: 4.05 × 10^12^ GC/mL as control) were microinjected into the left AIC (anteroposterior (AP) + 3.72 mm to bregma, mediolateral (ML) ±4.4 mm to midline suture; dorsoventral (DV) –5.6 mm to skull surface) or the left PIC (AP + 0.48 mm, ML + 5.6 mm, DV –7.4 mm) (Fig. 2B).

For pharmacogenetic inhibition of global AIC or PIC neuronal activity, rAAV2/9-hSyn-hM4Di-mCherry (titer: 6.96 × 10^12^ GC/mL) or rAAV2/9-hSyn- mCherry (titer: 6.25 × 10^12^ GC/mL as control) were bilaterally microinjected into the AIC (Fig. 3B) or PIC, at the above coordinates, 200 nL per side.

For selective pharmacogenetic inhibition of the glutaminergic AIC or PIC neurons, rAAV2/9-CaMKIIα-hM4Di-mCherry (titer: 5.89 × 10^12^ GC/mL) or rAAV 2/9-CaMKIIα-mCherry (titer: 5.04 × 10^12^ GC/mL as control) were bilaterally microinjected into the AIC or PIC (Fig. 4B) (coordinates as above), 200 nL per side.

For selective inhibition of the glutaminergic AIC neurons with projections to the PrL, either rAAV2/9-EF1α-DIO-hM4Di-mCherry (titer: 2.42 × 10^12^ GC/mL) or rAAV2/9-EF1α-DIO-mCherry virus (titer: 2.71 × 10^12^ GC/mL as control) was microinjected bilaterally into the AIC, 150 nL per side. Moreover, rAAV2/retro-CaMKIIα-Cre (titer: 6.62 × 10^12^ GC/mL) was bilaterally microinjected into the PrL (AP + 3.1 mm, ML ±0.70 mm, DV –4.30 mm), 200 nL per side (Fig. 6B).

### Optical Fiber Implantation

After virus injection, the optical fibers (optical fiber with 200 µm core diameter and ceramic ferrules with 2.50 mm diameter, Thorlabs) for photometric recording of neuronal activity were implanted in the brain at 50 µm above the viral injection sites in the unilateral AIC or PIC and were fixed with dental cement. Animals were allowed to recover for 4 weeks.

### Fiber Photometry

To record bilateral AIC and PIC glutaminergic neuronal activity during itch, rats received an intradermal injection of 5-HT unilaterally into the nape. During recording, both scratching behaviors and fluorescence signals in the ipsilateral AIC or PIC were simultaneously recorded by a fiber photometry system for 30 min after 5-HT injection. The contralateral fluorescence signals in the AIC or PIC were recorded by repeating this procedure when 5-HT was contralaterally administrated into the nape. A 488 nm laser (blue) was used to excite fluorescence signals. Moreover, a 405-nm laser (purple) was concurrently provided to isolate the movement-corrected signals from the channel. To minimize the bleaching of jGCaMP6s and EGFP, the laser power was adjusted to a low level of 20–40 µW at the tip of the optical fiber.

The fluorescence signals were analyzed offline with the built-in software of the fiber photometry system. The formula (F − F0)/F0 was used to calculate the change of the fluorescence values (ΔF/F), in which F indicates the fluorescence value at each time point during the period from −4 s to 10 s and F0 indexes the baseline median of the fluorescence value during the period from −4 s to −2 s, relative to the onset of each scratching train. To analyze the fluorescence changes across the itch behaviors, the average ΔF/F during the early (1–5 s), and later period (6–10 s), relative to the onset of each scratching train were calculated and finally averaged from all scratching trains for each animal and plotted as heatmaps (Fig. 2).

### Pharmacogenetic Manipulations

To pharmacogenetically inhibit the neuronal activity in the AIC or PIC, clozapine-N-oxide (CNO; 4 mg/kg, diluted in 5% DMSO, i.p.) was administered to rats having expressed hM4Di-mCherry or mCherry. Thirty minutes after the CNO injection, behavioral tests for itch, pain, or CPA [20, 21] were conducted.

### Itch-related Scratching Behavior Tests

Before itch-induced scratching tests, a small magnet was attached to one of the rat’s hind limbs. Each rat was placed in a transparent plastic chamber (40 cm long, 40 cm deep, and 60 cm high) and allowed to move freely. The scratching behaviors were monitored by a digital video camera fastened to the roof of the chamber and recorded by a magnetic induction method. At first, a 15-min baseline of scratching behavior was recorded. Then, the rat was gently removed from the chamber and 5-HT (5.00 mmol/L, 50 µL) or compound 48/80 (65 mmol/L, 50 µL) was injected intradermally in the unilateral nape ipsilateral to the hind limb with the small magnet. Scratching behaviors were continuously recorded for 30 min after pruritogen administration. A custom-written software was used to automatically analyze the itch-scratching data. Details and criteria to identify the scratching behaviors have been described previously [38, 43].

During itching, rats repeatedly moved their unilateral hind limb attaching with a small magnet to scratch the injection site. This action induced robust inductive coil voltage fluctuations. A 16-channel amplifier (AM Systems) was used to amplify and digitalize (sampling rate of 1000 Hz) the voltage signals. The amplified signals were bandpass filtered (3−100 Hz) and recorded with a data acquisition system (AD Instruments). Both the cumulative number and duration of scratching trains during the 15 min (baseline) and 30 min (after injection) were calculated for analysis. Data for each 5 min were averaged and analyzed.

### Conditioned Place Aversion (CPA)

Animals were injected with rAAV2/9-CaMKIIα-hM4Di-mCherry or rAAV2/9- CaMKIIα-mCherry (control) into the bilateral AIC. Four weeks after the virus injection, the CPA tests were applied using an unbiased, counterbalanced three-compartment conditioning apparatus and conducted under red light and sound-attenuated conditions [20, 21]. Each chamber had a unique combination of visual properties (one side had black walls, whereas the other side had black and white striped walls). Behavioral activity in each compartment was monitored and recorded with a video camera and analyzed using SA213 Conditioned Place Aversion v 1.0.

Itch-related CPA measurements were made on five successive days. In the pre-phase (day 1), rats were allowed to freely explore the entire apparatus for 15 min, beginning from the middle chamber. Animals with a significant bias toward either chamber ((t2–t1)/t1 >25%, where t represents the duration of animal stay in each chamber, t2 > t1) were excluded from subsequent experiments. The place aversion behaviors were conditioned during the acquisition phase (day 2 to day 4), with two conditioning trials on each day (morning session *versus* afternoon session) and a total of six acquisition trials. The left and right chambers were itch-unpaired and itch-paired, respectively, during successive conditioning days. On the morning of day 2, rats were intradermally injected with 50 µL saline into the unilateral nape, and restricted to the left chamber for 30 min. In the afternoon, rats received an intradermal injection of 5-HT (5.00 mmol/L in sterile saline) and were confined to the right chamber for 30 min. On day 3, rats received itch-paired training (injection with 5-HT) in the morning and itch-unpaired training (injection of saline) in the afternoon. On day 4, it received itch-unpaired training in the morning and itch-paired training in the afternoon.

On day 5 (post-phase), rats were given CNO (4 mg/kg) to selectively inhibit AIC glutaminergic neurons. Thirty minutes after the CNO injection, the rats were allowed to freely explore all three chambers for 15 min. Durations of stays in each chamber were recorded and analyzed (Fig. 5). The preference for the itch-paired chamber was calculated by the ratio of the time an animal spent in the itch-paired chamber to that in the itch-unpaired chamber.

### Pain-related Wiping Tests

A digital video camera was used to record pain-related behaviors. At first, the baseline of behavioral activity was recorded for 15 min. To evoke pain-related behaviors, the allyl isothiocyanate (AITC, 10% / 25 µL diluted with 7% Tween80, W203408, Sigma) was administered intradermally into the cheek [44]. Then the behaviors were recorded for 30 min after the AITC injection.

Digital video data were evaluated by two observers blinded to the behavioral procedures. The pain behavior was defined as a caudal-to-rostral wiping movement by the unilateral forelimb across the 5-HT injected location. Grooming with bilateral forelimbs and scratching with the hindlimb were excluded. Wipings are recognized as the effective measurement of pain-related behaviors [45]. The number of wipes was scored by the observers.

### Balance Beam Tests

A long beam (2 m × 20 mm) with a flat surface was used to examine the changes in motor ability. The beam was placed 50 cm above the floor, with a light at one end to provide aversive stimulation, and a black plastic box at the other end to motivate animals to cross the beam.

The balance beam tests were implemented on three successive days. In the first 2 days, rats were trained to cross the beam three times at minimal 10-min intervals. On day 3, the durations of three successful tests in which the rat crossed the beam without halt were averaged.

### Open Field Test (OFT)

The locomotor activity was evaluated by an OFT. Rats were initially placed in the center of the testing box (60 cm × 60 cm × 60 cm) and videotaped individually. The center area was defined as the centric 30 cm × 30 cm. Total travel distance and average speed were recorded in a 10-min period. The movement tracks of the rats were automatically analyzed by the software (XR-XZ301, Xinruan, China).

### Immunohistochemistry and Immunofluorescence

To detect insular neuronal c-fos expression and verify the expression of target genes, rats were given 5-HT, compound 48/80, or saline by intradermal injection (50 µL) into the unilateral nape before behavioral tests. Sixty minutes after the itch behavioral test or at the end of pharmacogenetic or fiber photometry experiments, rats were deeply anesthetized with an overdose of 3% pentobarbital sodium (i.p.) and perfused transcardiacally with physiological saline, followed by 4% paraformaldehyde (PFA; pH 7.4). The brain was removed and stored in 4% PFA (4°C, 24 h). The brain was then dehydrated successively in 10%, 20%, and 30% sucrose solutions for 24 h, 24 h, and 48 h, respectively. Coronal sections (30 µm thick) were cut on a freezing microtome. The sections were stored in cold PBS (0.01 mol/L, pH 7.4). Sections were placed in PBST (PBS + 0.3% Triton X-100) with 2% normal bovine serum albumin for 1 h, then incubated with primary antibody (4°C, 24 h, rabbit anti-c-Fos 1:500; mouse anti-NeuN 1:500; mouse anti-CaMKIIα 1:100). Then the sections underwent three wash steps for 10 min each in PBST, followed by 2 h incubation with secondary antibody (goat anti-rabbit conjugated to AlexaFluor488, 1:500; goat anti-mouse conjugated to AlexaFluor568, 1:500; goat anti-mouse conjugated to AlexaFluor488, 1:500, Invitrogen). Finally, the sections were washed with PBST (once, 10 min) and incubated for 10 min with DAPI (1:2000, D9542, Sigma-Aldrich). The sections then underwent three more wash steps of 10 min each in PBST, followed by mounting and coverslipping on microscope slides. Confocal fluorescence imaging with the antibodies and the fluorescent proteins were acquired using a slide view VS200 (Olympus, Japan). Coronal sections from 3 rats (2 sections per rat) were examined for statistical analysis.

### Histology

The extent of target gene expression and the placement of the optical fiber were examined by procedures as described previously [38, 43]. In brief, the deeply anesthetized rats were perfused; their brains were removed, stored, dehydrated, and coronally sectioned at 30 µm thick. The collected sections were washed with PBS (0.01 mol/L, pH 7.4) for 10 min and incubated with DAPI (1:2000) for another 10 min, followed by an additional three wash steps of 10 min each in PBS. Histological images were acquired by an Olympus BX53F fluorescence microscope (Japan) or an Olympus VS200 virtual slide system (Japan).

### Statistical Analysis

Data are expressed as the mean ± standard error (SEM). The statistical significance was determined by two-way ANOVA with repeated measures followed by the Tukey *post hoc* test or by two-tailed unpaired Student’s *t*-test using the SPSS for Windows package (v. 25.0). A value of *P* <0.05 was considered statistically significant.

## Results

### Both AIC and PIC Neurons Are Activated During Acute Itch

**Fig. 1:**
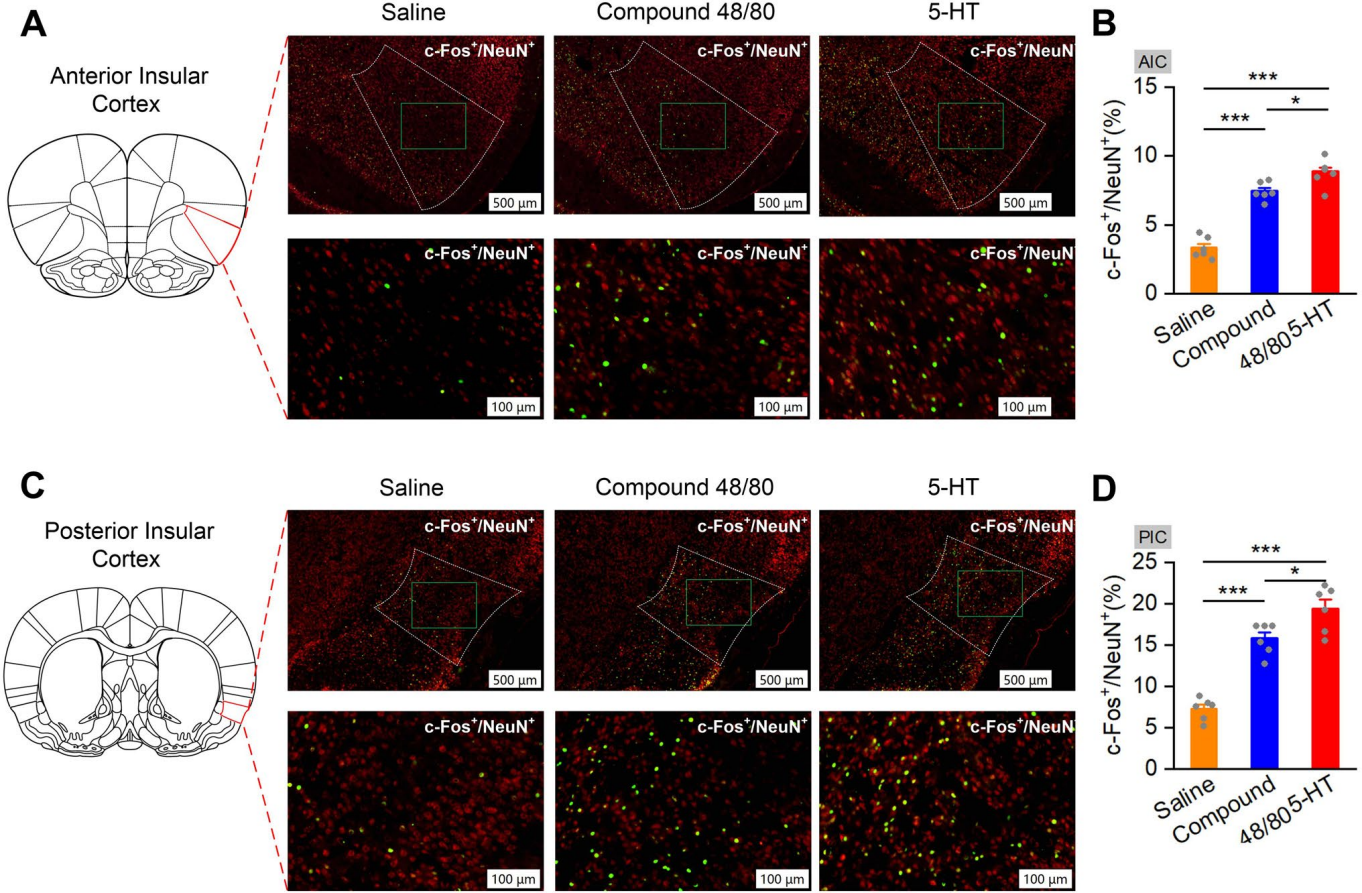
Percentages of c-Fos-positive cells are significantly increased in the AIC and PIC after pruritogen injection. **A** and **C** Representative immunohistochemical staining images of c-Fos-positive cells in the AIC (**A**) and PIC (**C**) after saline, compound 48/80, or 5-HT injection. Scale bars, 500 μm (upper panels) and 100 μm (lower panels). **B** and **D** Percentages of c-Fos-positive cells in the AIC (**B**, *n* = 3 rats per group, two sections per rat) and PIC (**D**, *n* = 3 rats per group, two sections per rat) are significantly increased after pruritogen injection. Data are shown as the mean ± SEM. One-way ANOVA followed by the LSD *post hoc* test. N.S., no significant difference, *P < 0.05, **P < 0.01, ***P < 0.001.

To assess the role of the AIC and PIC in itch processing, we checked the c-fos expression in the AIC and PIC after 5-HT and compound 48/80 administration (Fig. 1). Immunohistochemistry results indicated that the number of c-Fos positive (c-Fos^+^) neurons in the AIC and PIC significantly increased after 5-HT or compound 48/80 injection compared with the saline group (*F*_(2, 17)_ = 69.935, 5-HT: *P* <0.001, compound 48/80: *P* <0.001 for AIC; *F*_(2, 17)_ = 53.626, 5-HT: *P* < 0.001, compound 48/80: *P* <0.001 for PIC).

**Fig. 2:**
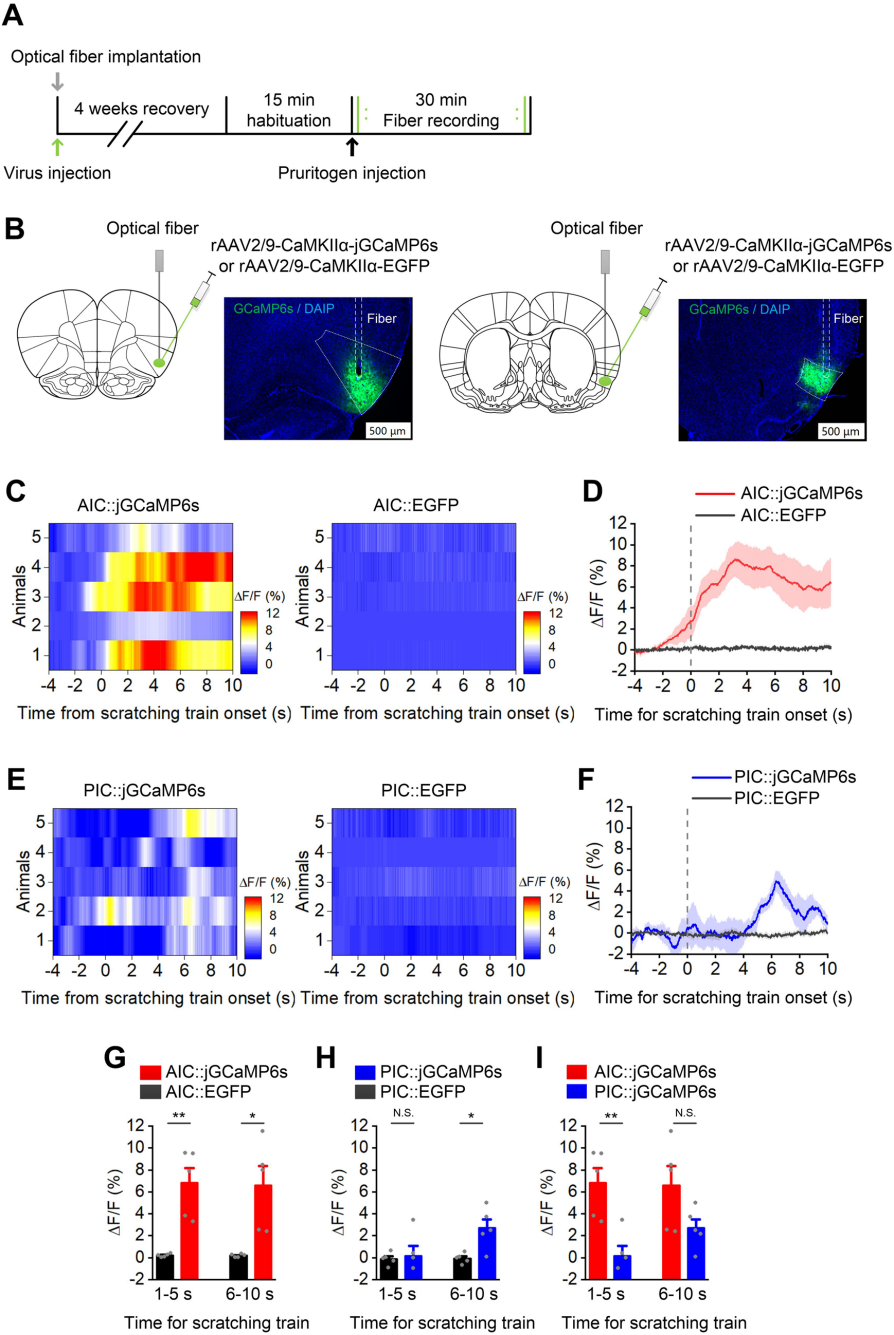
The activity of glutaminergic AIC and PIC neurons increases during 5-HT-induced itch. **A** Experimental timeline for virus injection and fiber recording.** B** Schematic showing the virus injection and optical fiber implantation into the AIC (left) and PIC (right). Scale bars, 500 μm. **C** and **E** Heatmaps showing the average fluorescence change in the unilateral AIC and PIC in the EGFP group and the jGCaMP6s group (*n* = 5 per group, each row represents an individual animal). **D** and **F** Average fluorescence change in unilateral AIC and PIC in the jGCaMP6s group and the EGFP group (*n* = 5 per group). Thick lines indicate the mean and shaded areas indicate the SEM. **G**–**I** Fluorescence signal recorded from the unilateral AIC and PIC during 5-HT-induced itch-related scratching (*n* = 5 per group). Comparison of the mean ΔF/F (left: 1−5 s, right: 6−10 s) between the jGCaMP6s and EGFP groups (**G** and **H**). Comparison of the mean ΔF/F (left: 1−5 s, right: 6−10 s) between the AIC::jGCaMP6s and PIC::jGCaMP6s groups (**I**). Each data point represents an individual animal. Data are shown as the mean ± SEM. Unpaired Student’s *t*-test, N.S., no significant difference, *P <0.05, **P <0.01, ***P <0.001.

To further confirm the activation of the AIC and PIC neurons and analyze the temporal relationship between insular neuronal activation and scratching behavior during itch-scratching, fiber photometry of intracellular Ca^2+^ signals in the AIC and PIC glutaminergic neurons was applied during 5-HT-induced itch (Fig. 2A). We microinjected rAAV expressing a Ca^2+^ indicator (jGCaMP6s) or EGFP under control of the CaMKIIα promoter (rAAV2/9-CaMKIIα-jGCaMP6s or rAAV2/9-CaMKIIα- EGFP) into the left AIC or PIC. Optic fibers were implanted above the injection sites (Fig. 2B). Four weeks later, fluorescence signals were recorded after ipsilateral and contralateral injections of 5-HT (2 days). We found that bilateral fluorescent signals of both AIC and PIC glutaminergic neurons significantly increased during 5-HT-induced itching (Fig. 2G and H show the ipsilateral result. *P* = 0.001 for 1−5 s and *P* = 0.007 for 6−10 s in AIC; *P* = 0.813 for 1−5 s and *P* = 0.011 for 6−10 s in PIC). Notably, the fluorescent signal of the AIC was significantly higher and occurred earlier than that of the PIC (Fig. 2I, P = 0.004 for 1−5 s; *P* = 0.083 for 6−10 s), and the elevation preceded the scratching behavior by ~1−2 s in the AIC (Fig. 2D), which indicates possible involvement of AIC neuronal activation in itch sensation or the aversive emotion accompanying itching.

### Nonselective Inhibition of the AIC Neurons Reduces 5-HT-induced Scratching

**Fig. 3:**
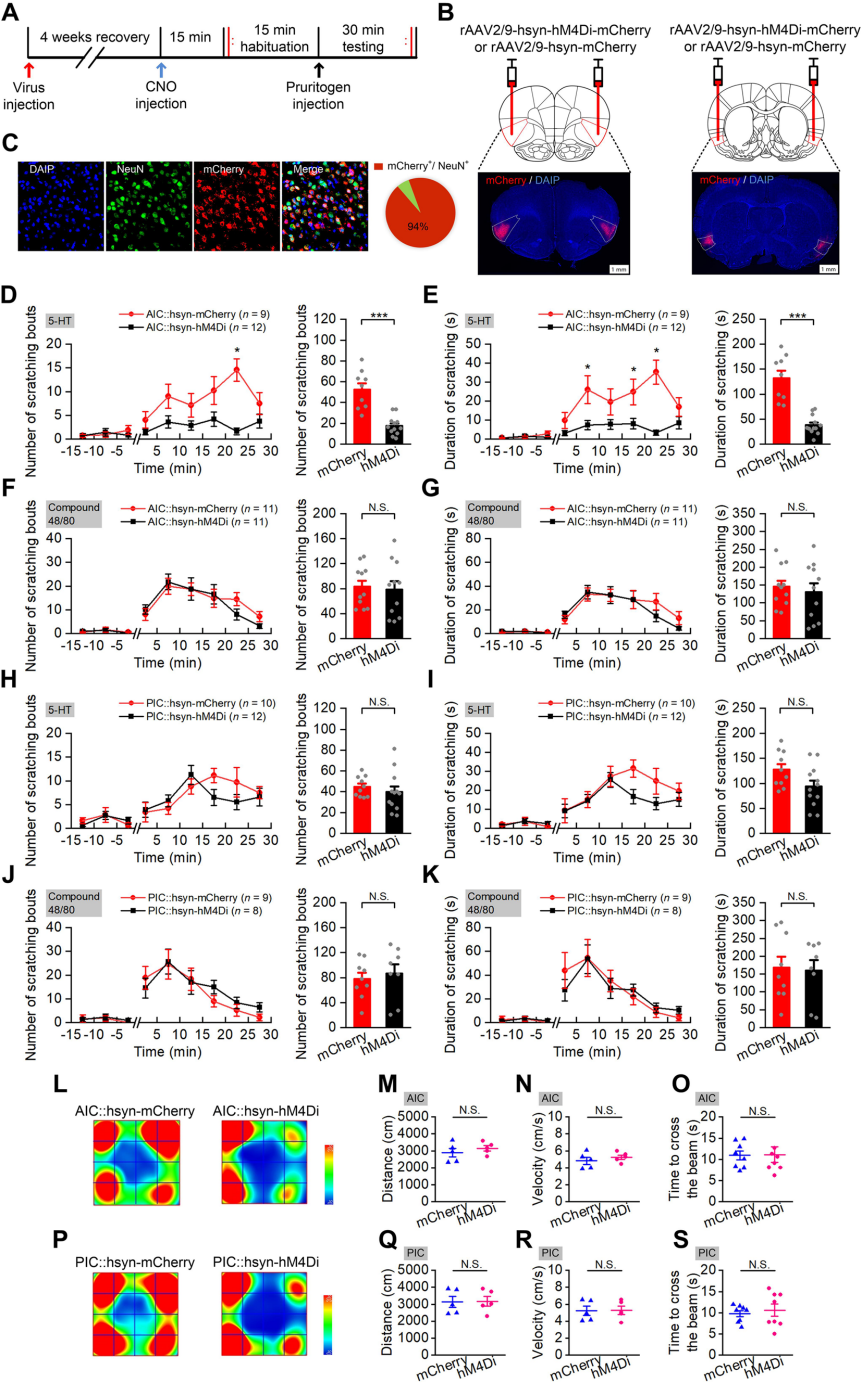
Non-selective inhibition of neurons in the AIC, but not the PIC, significantly decrease 5-HT-induced scratching. **A** Experimental timeline for virus injection and behavioral testing. **B** Schematic showing bilateral injection of virus into the AIC and PIC, Scale bar, 500 µm.** C** Representative images showing cell-specific hMD4i-mCherry expression (red) in neurons (green); statistics of expression in the neurons (*n* = 7 rats). **D**−**K** Pharmacogenetic inhibition of AIC neurons significantly decreases 5-HT-, but not compound 48/80-evoked scratching (**D**−**G**) and pharmacogenetic inhibition of neurons in the PIC does not significantly affect either 5-HT- or compound 48/80-evoked scratching (**H**−**K**). (*n* value for the AIC::hM4Di group and the AIC::mCherry group is shown in each Fig.). The number of scratching bouts during testing (**D**, **F**, **H**, and **J**). (Left) The number of scratching bouts for each 5 min during 15 min habituation and 30 min testing; (Right) the total number of scratching bouts during 30 min testing. The cumulative duration of scratching bouts during testing (**E**, **G**, **I**, and **K**). (Left) The cumulative duration of scratching bouts for each 5 min during 15 min habituation and 30 min testing; (Right) the total cumulative duration during 30 min testing. Data are shown as the mean ± SEM. (Left) Two-way ANOVA with repeated measures followed by Tukey’s *post hoc* test; (Right) unpaired Student’s *t*-test. N.S., no significant difference, ***P <0.001. **L**−**N**, **P**−**R** Pharmacogenetic inhibition of AIC or PIC neurons does not significantly affect the distance moved and velocity in the OFT (*n* = 5 per group). Representative heat maps (**L**, **P**) showing the location in OFT tests of mCherry control (left) and hM4Di (right) in the AIC (**L**) group and the PIC (**P**) group; total distance moved in the OFT (**M**, **Q**); movement velocity in OFT (**N**, **R**). Data are shown as the mean ± SEM. Unpaired Student’s *t*-test. N.S., no significant difference. **O**, **S** Pharmacogenetic inhibition of AIC or PIC neurons does not significantly affect the time to cross the balance beam (*n* = 8 per group). Data are shown as the mean ± SEM. Unpaired Student’s *t*-test. N.S., no significant difference.

Although we had revealed that the AIC and PIC neurons were activated during itch, direct evidence to confirm the role of the insula in itch regulation was still insufficient. To functionally identify the involvement of the AIC and PIC neurons in the modulation of acute itch, we used the pharmacogenetic approach of designer receptors exclusively activated by designer drugs (DREADDs) (Fig. 3A). To conduct the pharmacogenetic manipulations on global AIC or PIC neurons, recombined adeno-associated virus (rAAV) encoding the inhibitory DREADD receptor hM4Di or control (rAAV2/9-hsyn-hM4Di-mCherry or rAAV2/9-hsyn-mCherry as control), were microinjected bilaterally into the AIC or PIC (Fig. 3B). Four weeks after virus injection, the expression of hM4Di-mcherry was confirmed by immunohistochemistry (Fig. 3C, 94% of NeuN-immunopositive cells expressed hM4Di-mcherry). The itch behavioral test was applied after administration of CNO (i.p.), and the OFT and balance beam test were applied to measure the motor coordination after pharmacogenetic inhibition of the AIC or PIC neurons.

The results indicated that global inhibition of the AIC neurons reduced the number and cumulative duration of scratching bouts elicited by 5-HT (Fig. 3D and E, F_(1, 19)_ = 33.262, *P* <0.001; *F*_(1, 19)_ = 42.182, *P* <0.001 for the number of scratching bouts, *P* <0.001 for the total duration of scratching bouts), but not compound 48/80 (Fig. 3F and G, F_(1, 20)_ = 0.076, *P* = 0.785 for number of scratching bouts, *P* = 0.785 for total number for scratching bouts; *F*_(1, 20)_ = 0.25, *P* = 0.623 for duration of scratching bouts, *P* = 0.623 for total duration of scratching bouts).

Global pharmacogenetic inhibition of the PIC neurons did not affect the number and cumulative duration of scratching bouts elicited by 5-HT (Fig. 3H and I, F_(1, 20)_ = 0.602, *P* = 0.447 for the number of scratching bouts, *P* = 0.447 for the total number of scratching bouts; *F*_(1, 20)_ = 4.117, *P* = 0.056 for the duration of scratching bouts, *P* = 0.056 for the total duration of scratching bouts), or compound 48/80 (Fig. 3J and K, F_(1, 15)_ = 0.246, *P* = 0.627 for number of scratching bouts, P = 0.627 for total number for scratching bouts; *F*_(1, 15)_ = 0.035, *P* = 0.855 for duration of scratching bouts, *P* = 0.855 for total duration of scratching bouts). In addition, the pain-related wiping responses were also measured. The results showed that inhibition of global AIC or PIC failed to affect the pain-related wiping (Fig. 7B and C, *P* = 0.503 for AIC, *P* = 0.926 for PIC). Pharmacogenetic inhibition of the AIC or PIC neurons did not affect the motor coordination (Fig. 3O and S, P = 0.941 for AIC, *P* = 0.622 for PIC), and did not significantly affect the distance moved and velocity in the OFT (Fig. 3M–R, P = 0.41 for AIC,* P* = 0.924 for PIC).

### Selective Inhibition of AIC Glutaminergic Neurons Significantly Reduces 5-HT-induced Scratching

**Fig. 4:**
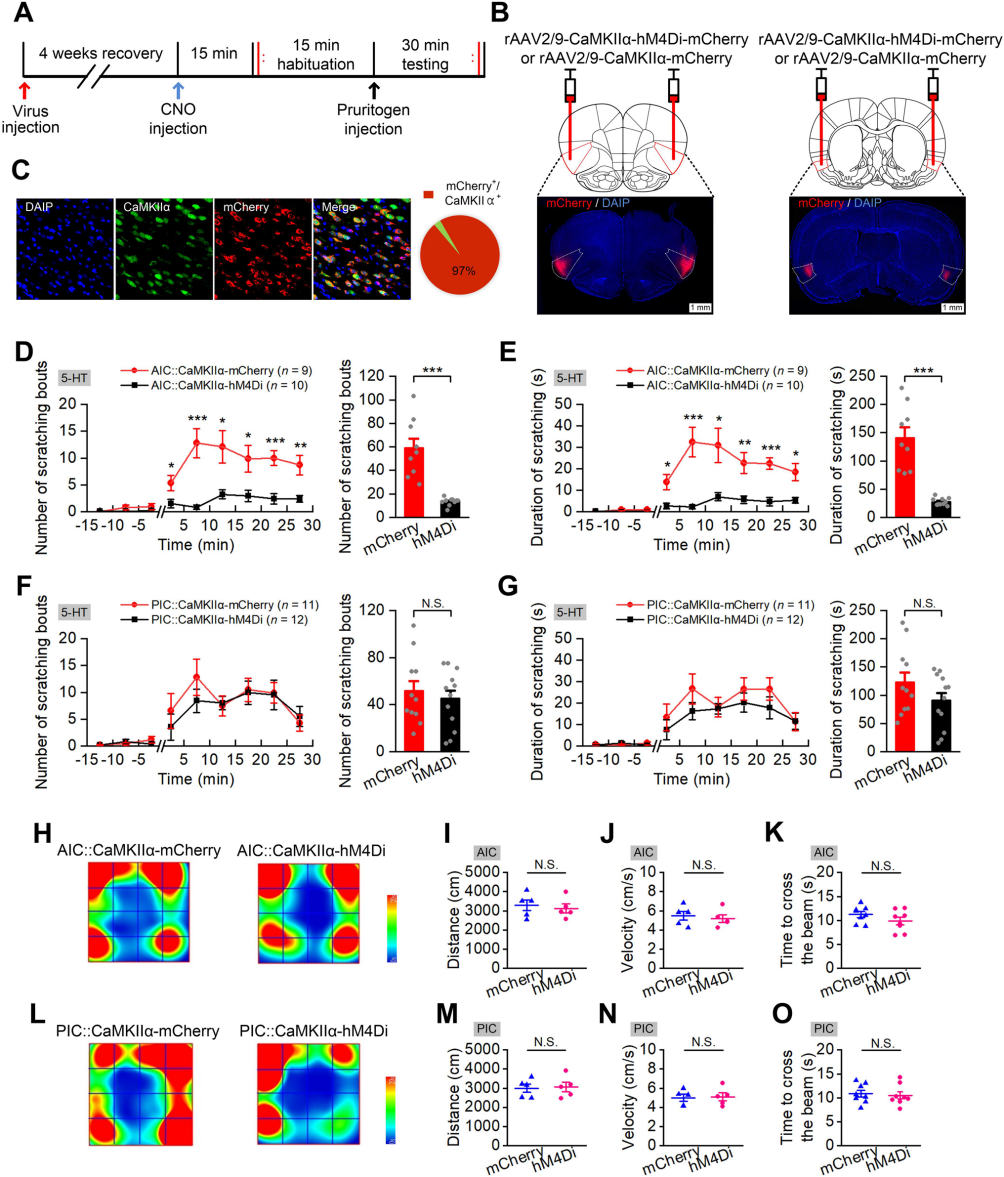
Selective inhibition of glutaminergic neurons in the AIC, but not the PIC, significantly decreases 5-HT-induced scratching. **A** Experimental timeline for virus injection and behavioral testing.** B** Schematic showing bilateral injection of virus into the AIC (left) and PIC (right); scale bar, 500 µm. **C** Representative images showing cell-specific hMD4i-mCherry expression (red) in neurons (green) and statistics of expression in the neurons (*n* = 7 rats). **D**−**G** Pharmacogenetic inhibition of glutaminergic neurons in the AIC, but not the PIC, significantly decrease 5-HT evoked scratching. The number of scratching bouts during testing (**D** and **F**). (Left) The number of scratching bouts for each 5 min during 15 min habituation and 30 min testing; (right) the total number of scratching bouts during 30 min testing. The cumulative duration of scratching bouts during testing (**E** and **G**). (Left) The cumulative duration of scratching bouts for each 5 min during 15 min habituation and 30 min testing; (right) the total cumulative duration during 30 min testing. Data are shown as the mean ± SEM. (left) Two-way ANOVA with repeated measures followed by Tukey’s *post hoc* test, (right) unpaired Student’s *t*-test. N.S., no significant difference, ***P <0.001. **H**−**J**, **L**−**N** Pharmacogenetic inhibition of glutaminergic AIC or PIC neurons does not significantly affect the distance moved and velocity in the OFT (*n* = 5 per group). Representative heat maps (**H**, **L**) showing spatial location in OFTs of mCherry control (left) and hM4Di (right) in the AIC (**H**) and PIC (**L**) groups; total distance moved in the OFT (**I**, **M**); movement velocity in the OFT (**J**, **N**). Data are shown as the mean ± SEM. Unpaired Student’s *t*-test. N.S., no significant difference. **K**, **O** Pharmacogenetic inhibition of the glutaminergic AIC or PIC neurons does not significantly affect the time to cross the balance beam (*n* = 8 per group). Data are shown as the mean ± SEM. Unpaired Student’s *t*-test. N.S., no significant difference.

To dissect the involvement of subpopulations of the AIC and PIC neurons in the modulation of acute itch, cell type-specific pharmacogenetic manipulations were used to inhibit glutaminergic neurons in both the AIC and PIC during 5-HT-induced itch (Fig. 4A). The results demonstrated that pharmacogenetic inhibition of AIC glutaminergic neurons reduced the number and the cumulative duration of scratching bouts elicited by 5-HT (Fig. 4D and E, F_(1,17)_ = 33.368, *P* <0.001 for number of scratching bouts, *P* <0.001 for total number for scratching bouts; *F*_(1, 17)_ = 39.655, *P* <0.001 for duration of scratching bouts, *P* <0.001 for total duration of scratching bouts). Nevertheless, inhibition of the PIC glutaminergic neurons had no significant effect on 5-HT-induced scratching behavior (Fig. 4F and G, F_(1, 21)_ = 0.346, *P* = 0.563 for number of scratching bouts, *P* = 0.563 for total number for scratching bouts; *F*_(1, 21)_ = 2.008, *P* = 0.171 for duration of scratching bouts, *P* = 0.171 for total duration of scratching bouts).

In addition, selective inhibition of glutaminergic neurons in the AIC, but not PIC, significantly decreased the pain-related wiping by the forelimb elicited by AITC (Fig. 7D and E, P = 0.02 for AIC, *P* = 0.869 for PIC). Pharmacogenetic inhibition of both AIC and PIC glutaminergic neurons did not affect motor coordination (Fig. 4K and O, P = 0.184 for AIC, *P* = 0.734 for PIC), and did not significantly affect the distance moved and velocity in OFT (Fig. 4I–N, P = 0.669 for AIC, *P* = 0.865 for PIC).

### Selective Inhibition of AIC Glutaminergic Neurons Blocks Itch-associated CPA

**Fig. 5:**
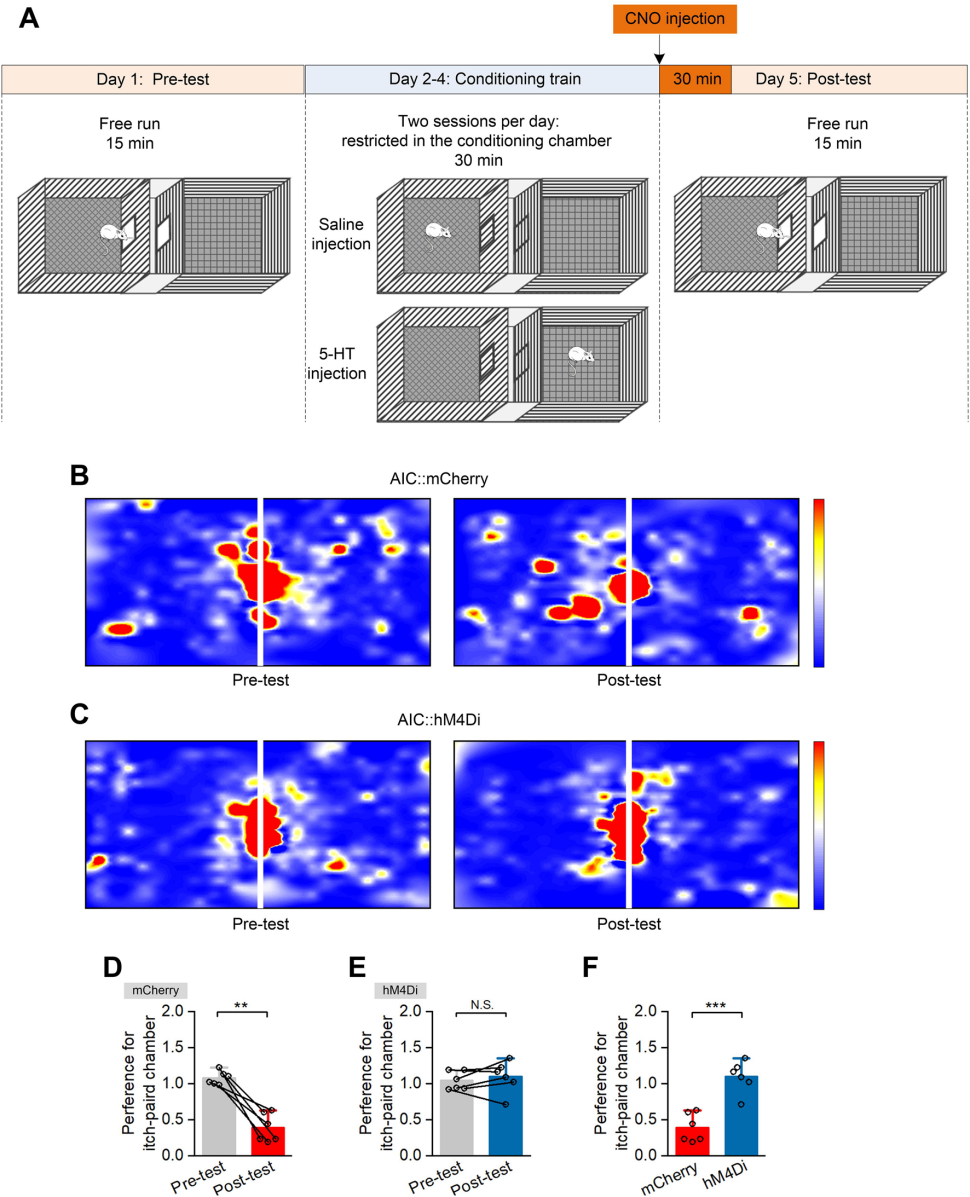
Selective inhibition of AIC glutaminergic neurons blocks itch-associated conditioned place aversion. **A** Schematic of the design of conditioned place aversion experiments. **B** and **C** Representative heat maps showing the spatial location in pre-test and post-test of the mCherry control group (**B**) and the hM4Di group (**C**). **D**−**F** Preference for the itch-paired chamber. Preference for itch-paired chamber (**D** and **E**) during pre-test and post-test of the mCherry group (**D**, *n* = 6) and the hM4Di group (**E**, *n* = 6). Comparison of preference for the itch-paired chamber (**F**) during post-test of the mCherry group and the hM4Di group. Data are shown as the mean ± SEM. Unpaired Student’s *t*-test, N.S., no significant difference, ***P <0.001.

To investigate the contributions of glutaminergic AIC neurons to mediating the aversive aspects of itch-scratching, the effects of selective pharmacogenetic inhibition of glutaminergic AIC neurons on CPA behavior were examined (Fig. 5A). During CPA pre-training on day 1, rats were allowed free access to both chambers for 15 min without administration of pruritogens, and animals that had spent similar times in both chambers in the inhibition (AIC::hM4Di) and control (AIC::mCherry) groups were selected for further experiments. After 3 days of CPA with 5-HT administration, control rats exhibited an evident avoidance of the itch-paired chamber, indicated by the shorter time spent in the itch-paired chamber during post-training than that during pre-training (Fig. 5B and D, P = 0.001). Interestingly, rats in the inhibition group did not show an aversive response to the itch-paired chamber, in which they spent time similar to that in the itch-paired chamber during pre- and post-training (Fig. 5C and E, P = 0.476). In addition, the preference for the itch-paired chamber during post-training was significantly lower in the control group than that in the inhibition group (Fig. 5F, P <0.001), suggesting that pharmacogenetic inhibition of glutaminergic AIC neurons effectively blocks the itch-associated CPA during the post-training test.

### Selective Inhibition of Glutaminergic AIC Neurons Projecting to the PrL Suppresses 5-HT-Evoked Scratching

**Fig. 6:**
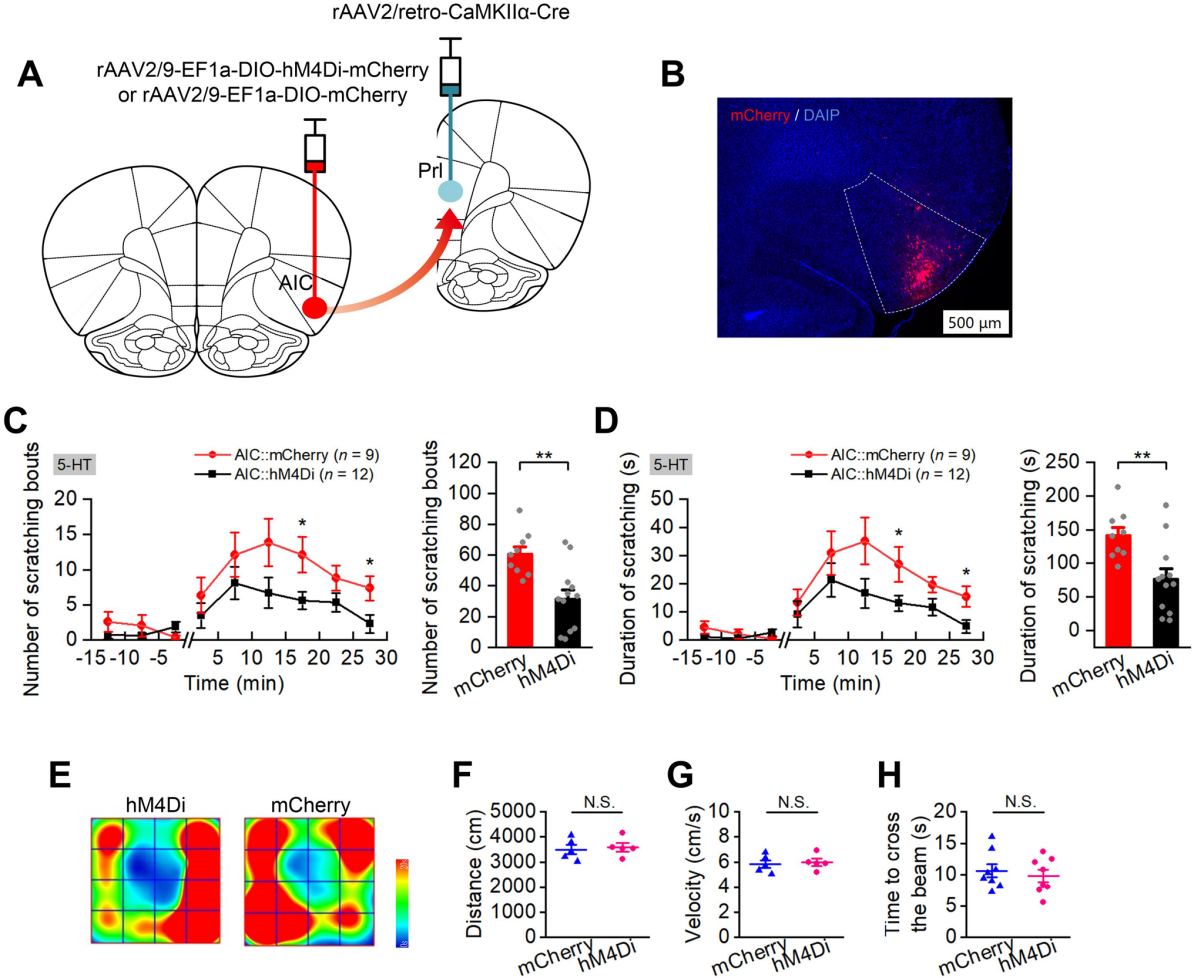
Selective inhibition of glutaminergic AIC neurons projecting to the Prl significantly suppresses 5-HT-evoked scratching. **A** Schematic of the experimental design for viral injection to inhibit the glutaminergic neurons in the AIC projecting to the Prl. **B** Representative image showing the expression of hM4Di-mCherry in the AIC; scale bar, 500 µm. **C** and **D** Pharmacogenetic inhibition of the glutaminergic AIC neurons projecting to the Prl significantly decreases 5-HT-evoked scratching (*n* = 9 for mCherry and *n* = 12 for the hM4Di group). The number of scratching bouts during testing (**C**). (Left) the number of scratching bouts for each 5 min during 15 min habituation and 30 min testing; (Right) the total number of scratching bouts during 30 min testing. The cumulative duration of scratching bouts during testing (**D**). (Left) The cumulative duration of scratching bouts for each 5 min during 15 min habituation and 30 min testing; (Right) the total cumulative duration during 30 min testing. Data are shown as the mean ± SEM. (left) Two-way ANOVA with repeated measures followed by Tukey’s *post hoc* test, (right) unpaired Student’s *t*-test. *P <0.05, **P <0.01. **E–G** Pharmacogenetic inhibition of glutaminergic AIC neurons projecting to the Prl does not significantly affect the distance moved and velocity in the OFT (*n* = 5 per group). Representative heat maps (**E**) showing the spatial location in OFTs of hM4Di (left) and mCherry control (right) groups; total distance moved in the OFT (**F**); movement velocity in the OFT (**G**). Data are shown as the mean ± SEM. Unpaired Student’s *t*-test. N.S., no significant difference. **H** Pharmacogenetic inhibition of glutaminergic AIC neurons projecting to the Prl does not significantly affect the time to cross the balance beam (*n* = 8 per group). Data are shown as the mean ± SEM. Unpaired Student’s *t*-test. N.S., no significant difference.

**Fig. 7:**
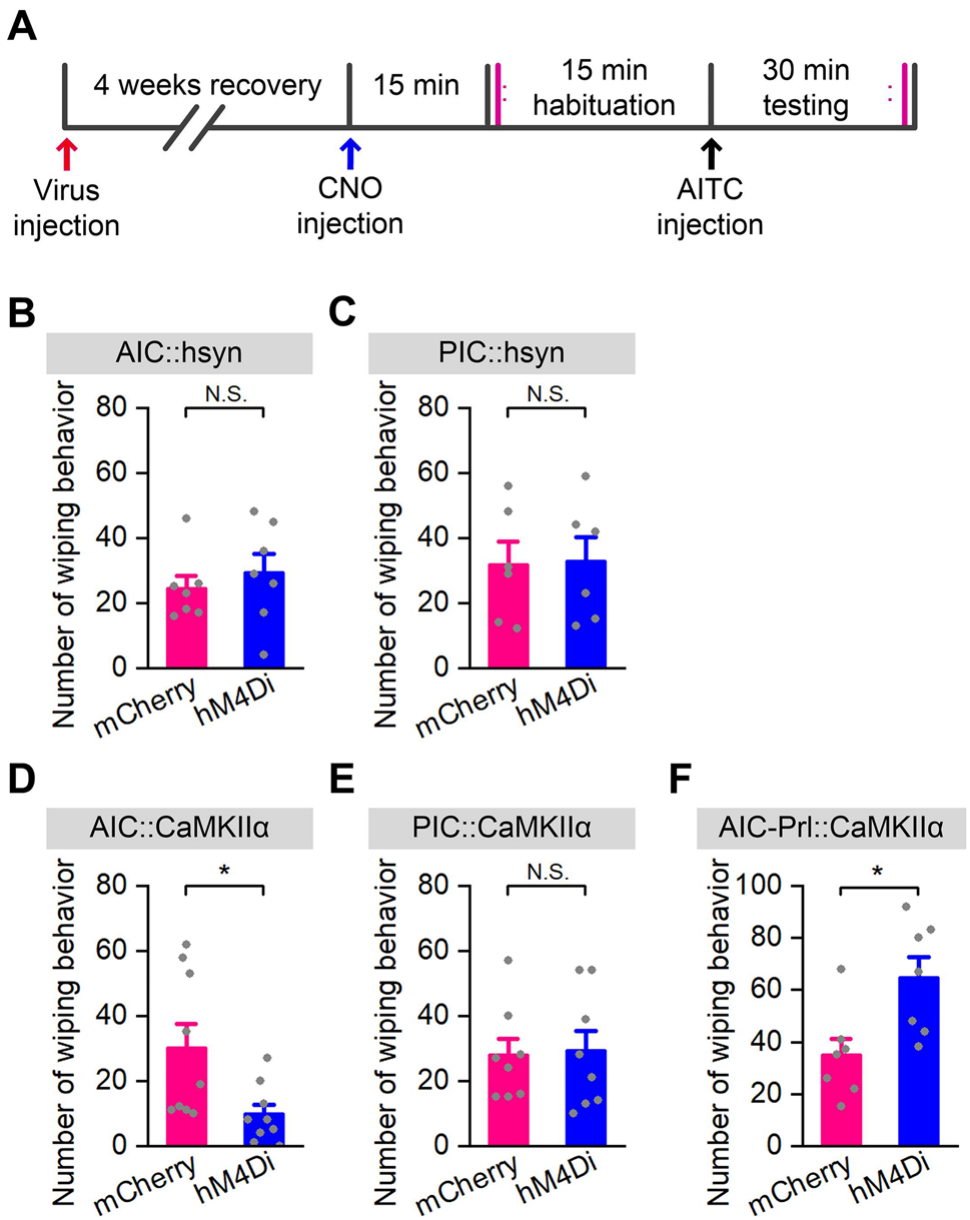
Effects of pharmacogenetic inhibition of AIC or PIC neurons on pain-related wiping. **A** Schematic of the design of pain-related wiping tests. **B**, **C** Pharmacogenetic inhibition of the AIC or PIC neurons does not significantly affect pain-related wiping (*n* = 7 for AIC::hM4Di group and AIC:: mCherry group, *n* = 6 for PIC::hM4Di group and PIC:: mCherry group). Data are shown as the mean ± SEM. Student’s *t*-test, N.S., no significant difference. **D**, **E** Pharmacogenetic inhibition of glutaminergic neurons in the AIC, but not the PIC, decrease pain-related wiping (*n* = 9 for AIC::hM4Di group and AIC::mCherry group, *n* = 8 for PIC::hM4Di group and PIC::mCherry group). Data are shown as the mean ± SEM. Student’s *t*-test, N.S., no significant difference, **P <0.01. **F** Pharmacogenetic inhibition of the AIC neurons projecting to the Prl increases pain-related wiping (*n* = 7 for hM4Di group, *n* = 7 for mCherry group). Data are shown as the mean ± SEM. Unpaired Student’s *t*-test, *P <0.05.

It has been reported in a series of functional imaging studies that the prelimbic cortex, a subregion of the prefrontal cortex closely associated with cognition, emotion, and motivation, is activated during itching [13, 46, 47]. Pharmacogenetic inhibition of the PrL reduces both 5-HT- and compound 48/80-induced scratching behaviors [31]. Given the verified role of glutaminergic AIC neurons in itch regulation in this study and the close anatomical connection between the AIC and PrL [48], we then assessed the regulatory effect of the AIC-PrL projections during itch processing. To selectively express hM4Di in glutaminergic AIC neurons descending to the PrL, a Cre-dependent AAV (rAAV2/9-EF1α-DIO-hM4Di-mCherry) and a retrograde AAV expressing Cre recombinase (rAAV2/retro-CaMKIIα-Cre) were bilaterally microinjected into the AIC and the PrL, respectively (Fig. 6A). Five weeks after virus injection, an itch behavior test was implemented to examine the effect of these neurons in regulating itch-scratching. The results showed that selective inhibition of the AIC-PrL projection reduced the number and cumulative duration of scratching bouts elicited by 5-HT (Fig. 6C and D, F_(1,19)_ = 12.847, *P* = 0.003 for number of scratching bouts, *P* = 0.002 for total number for scratching bouts; *F*_(1,19)_ = 9.787, *P* = 0.04 for duration of scratching bouts, *P* = 0.005 for total duration of scratching bouts). Conversely, inhibition of this circuit significantly increased the pain-related wiping with the forelimb elicited by AITC (Fig. 7F, P = 0.014). Pharmacogenetic inhibition of the AIC-PrL projection did not affect motor coordination (Fig. 6H, P = 0.571), and did not significantly affect the distance moved (Fig. 6F, P = 0.738) and velocity (Fig. 6G, P = 0.735) in the OFT test.

## Discussion

### The AIC Makes a More Important Contribution than the PIC in the Modulation of Itch

Although cumulative studies have identified the insula as a core region related to multiple brain functions and a series of psychiatric and neurological disorders [48–51], imaging studies on itch have yielded extensive correlations with the insular lobe [12, 16–18], the role and functional specificity of the IC in itch modulation remain ambiguous. Here, we demonstrated that bilateral inhibition of global AIC neurons or selective inhibition of the glutaminergic neurons in the AIC reduced the scratching behaviors induced by intradermal injection of 5-HT but not those induced by compound 48/80. Nevertheless, both inhibition of global PIC neurons and selective inhibition of the glutaminergic neurons in the PIC failed to affect itching-scratching behaviors induced by both 5-HT and compound 48/80. The current results provide preliminary evidence that the AIC is involved in the regulation of 5-HT-induced itch.

Most imaging studies have indicated that the AIC, but not the PIC, are activated during the itching processes [16–18]. Given that the immunohistochemistry and fiber photometry results showed activation of PIC neurons during itching in the present study, the involvement of the PIC in itch regulation cannot be excluded. Conservative speculation suggests that the role of the AIC is far more crucial than that of the PIC in itch regulation. It should be noted that neuronal activation of the PIC occurred slightly later than that of the AIC. This differential temporal pattern between the AIC and PIC suggests that they might be involved in different aspects of itch processing.

Differences between AIC and PIC activation during pain processes have been extensively addressed by imaging studies, in which most of the results indicated that pain stimulation activates the AIC rather than the PIC [52–56]. The current results show that inactivating the glutaminergic neurons in the AIC, but not the PIC, significantly reduced pain responses, supporting the previous imaging findings. However, global inhibition of the AIC in this study failed to affect pain behavior. Since only nociceptive responses to mustard oil were examined, a reasonable interpretation of the AIC involvement in pain needs more evidence.

### The AIC May be Involved in Itch Regulation Through the Mediation of Itch-Associated Aversion

Recent experimental studies have revealed that specific neuronal subtypes in the PAG and VTA of the midbrain are responsible for mediating negative or positive itch-scratching emotions [20, 21]. Here, we found that pharmacogenetic inhibition of glutaminergic AIC neurons effectively blocked itch-associated CPA behavior, supporting the roles of the insula in processing itch-associated aversive emotion [12, 50, 57–59]. The insula has been previously acknowledged to be critical in linking pain sensation with aversive emotional responses. Patients with insula lesions can recognize pain but lack an appropriate aversive emotional response [50, 57–59]. Our current results support the conclusion that the AIC is involved in itch regulation, at least partially, through its mediation of itch-associated aversion. As multiple brain regions, such as the PAG [21], VTA [20], and amygdala [23], are involved in itch-associated aversion, the AIC may function together with these emotion-regulatory brain areas to modulate the negative emotion accompanying itch.

### The AIC-PrL Projection is Involved in the Modulation of Itch

The AIC reciprocally connects with frontal brain regions, including the medial prefrontal cortex (mPFC) [48], a key region implicated in cognitive, emotional, and executive functions. The PFC has also been identified as closely associated with itch regulation in functional imaging studies [12–18]. Inhibition of the PrL, a subregion of the mPFC, reduced both 5-HT- and 48/80-induced itch in rats [31]. Based on these previous findings and the close anatomical connection between the PrL and AIC [48], we examined the role of the AIC-PrL projection in itch processing. The results revealed that selective inhibition of AIC neurons projecting to the PrL reduced itch scratching but increased pain swiping behaviors, suggesting its differential involvement in the modulation of itch and pain. Unusually, inhibition of the total glutaminergic AIC neurons and the AIC glutaminergic neurons projecting to the PrL showed opposite effects on pain regulation. Perhaps different subpopulations of glutaminergic neurons in the AIC are differentially involved in pain modulation.

## Limitations and Conclusions

Several limitations in this study should be emphasized: (1) Only chemogenetic inhibition was used in this study during cellular interventions. As the negative result obtained using chemogenetic inhibition could be due to a lack of coupling between the chemogenetic receptors and endogenous signaling molecules, the exact role of the PIC in itch regulation is still inconclusive. (2) To simplify behavioral tests in this study, CPA behaviors were only measured in CaMKII-hM4Di-injected rats and not in hSyn-hM4Di-injected rats, and only one projection from the AIC was examined for its role in itch regulation. (3) The pain response caused by the injection of AITC into the cheek is not a classic pain behavior paradigm, and only one test of nociception provides insufficient evidence to conclude, so here we comment mainly on the involvement of the AIC in itch, rather than pain processes. Our preliminary results support a cautious conclusion that the AIC and its projection to the PrL play a critical role in regulating 5-HT- but not compound 48/80-induced itch. The roles of diverse insular neuronal subtypes and projections in modulating itch and the accompanying aversive emotion deserve further investigation.
